# Cartilage mechanical responses during gait as in silico biomarkers for medial knee OA progression

**DOI:** 10.1038/s41598-025-19371-2

**Published:** 2025-10-09

**Authors:** Yixuan Zhang, Bryce A. Killen, Ikram Mohout, Miel Willems, Frank P. Luyten, Sabine Verschueren, Seyed Ali Elahi, Ilse Jonkers

**Affiliations:** 1 Department of Movement Sciences, KU Leuven, Leuven, Belgium; 2 Skeletal Biology and Engineering Research Center, Department of Development and Regeneration, KU Leuven, Leuven, Belgium; 3 Department of Rehabilitation Sciences, KU Leuven, Leuven, Belgium; 4 Division of Biomechanics, Department of Mechanical Engineering, KU Leuven, Leuven, Belgium

**Keywords:** Osteoarthritis, Cartilage, Biomedical engineering

## Abstract

Osteoarthritis (OA) is a common degenerative whole joint disorder characterized by articular cartilage breakdown, mostly often in the knee’s medial compartment. Traditional attempts to predict structural progression have focused on biochemical markers from blood, synovia, or urine; however, these do not capture internal mechanical environments that drives cartilage degeneration. In silico modelling enables non-invasive, patient-specific estimation of internal joint loading, including contact pressure, compressive strain, fibril strain and maximum shear strain, as potential mechanical biomarkers for OA progression. This study developed a novel MSK-FE workflow linking gait kinematics to contact pressures and cartilage tissue strains. Subjects showing medial OA progression over 2 years exhibited elevated medial compartment contact pressures and a posterolateral location shift at baseline. Unsupervised k-means clustering of cartilage tissue strain histograms further distinguished progressors from non-progressors and controls. These results demonstrate the potential of computationally efficient mechanical biomarkers to identify patients at risk of OA progression, supporting early and targeted preventative treatment strategies.

**Supplementary Information:**

The online version contains supplementary material available at 10.1038/s41598-025-19371-2.

## Introduction

Osteoarthritis (OA) is the most common chronic joint disease, affecting over 240 million people worldwide^1^ and projected to 1 billion cases by 2050. It is characterized by inflammation, loss of joint function^2^ and cartilage degeneration^3^. Current OA treatments primarily focus on symptom relief and functional improvement^4,5^ but cannot restore cartilage to its original healthy state.

The rate of OA progression varies widely among patients^6^. Early interventions can improve outcomes and delay the needs for joint replacement^7^particularly in those at risk of fast progression. Identifying such patients early is therefore, critical to enable timely and effective management strategies to slow disease advancement and preserve joint function.

Mechanical loading is a key contributor to articular cartilage degeneration^3^. While, in vitro studies have shown that changes in loading magnitude and location induce cartilage microstructural damage and disturb chondrocyte homeostasis^8–10^ in silico modelling enables these in vitro insights^11^ to be extended in vivo. Parameters such as contact pressure and compressive strain reflect cartilage loading, while cartilage mechanical responses (e.g. fibril strain and maximum shear strain) are directly associated with cartilage microstructural degeneration (e.g. collagen degradation and proteoglycans depletion)^11–14^. Together these parameters represent candidate mechanical biomarkers of OA progression.

Compared to traditional biomarkers or clinical observation, in silico approaches provide a non-invasive and patient-specific means to estimate internal joint loading^15–18^ and cartilage tissue mechanical responses^14,19–25^ during gait. Musculoskeletal (MSK) modelling combined with 3D motion capture, can quantify patient-specific contact pressures^16–18^ while finite element (FE) models incorporating complex fibril-reinforced biphasic cartilage material properties, translate these loading into tissue-level mechanical responses, such as compressive strain, fibril strain and maximum shear strain^14,19–25^ presenting promising mechanical biomarkers for OA progression.

Previous studies^17,19^ including our own^14^ have demonstrated that this framework can detect OA-related differences in cross-sectional comparisons. The rationale of this study, is therefore to test whether an MSK-FE workflow applied to a unique longitudinal historical data set^26,27^ can identify mechanical biomarkers that discriminate OA progressors, with the ultimate goal of supporting early screening of OA progression to facilitate timely preventative rehabilitation strategies.

## Methods

**Fig. 1:**
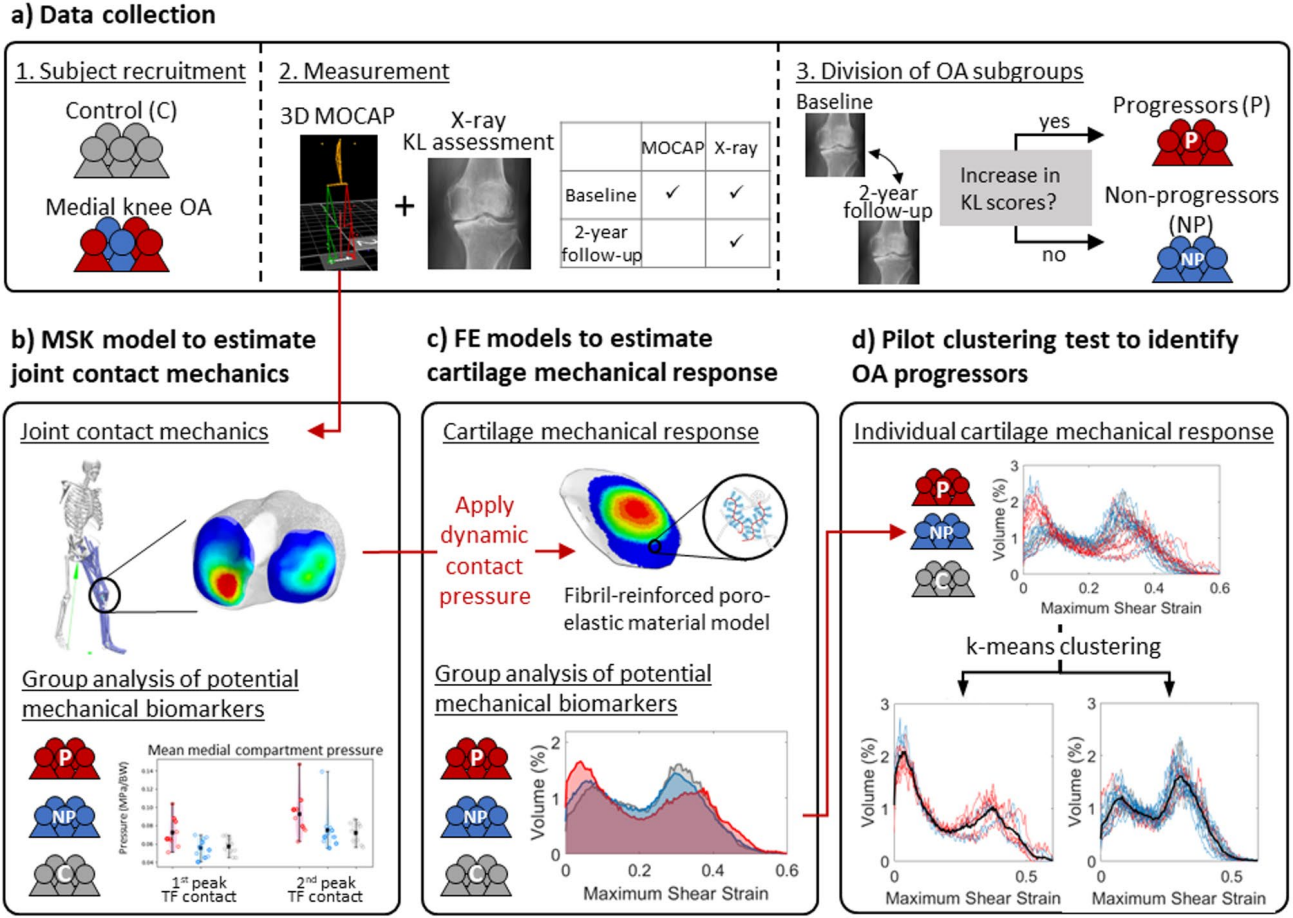
Study workflow. (**a**) Healthy controls (C) and patients with medial knee OA are recruited for 3D motion capture and x-ray to assess Kellgren-Lawrence (KL) scores of both knees. OA patients are divided into progressors (P) and non-progressors (NP) groups based on the change of KL score in the 2-year follow-up. (**b-c**) Joint contact mechanics are estimated by MSK modeling using 3D motion capture. The dynamic contact pressure of the stance phase estimated by MSK models is then applied to FE cartilage models. Group-level analysis of contact mechanics and cartilage tissue mechanical responses are performed to identify in *silico* mechanical biomarkers characteristic for OA progression. (**d**) A pilot clustering test uses cartilage mechanical responses estimated for individual subjects to identify OA progressors.

Figure 1 illustrates the study’s workflow, detailing the integration of gait analysis, MSK modeling, and FE simulations to identify mechanical cartilage tissue parameters that differentiate OA progressors from non-progressors and controls.

Data Collection.

This study is a secondary analysis of a prospective clinical study using a longitudinal dataset of healthy control and knee OA female participants^26,27^. Ethics approval was obtained from the local ethics committee UZ Leuven (Clinical trial number: S50534) - in accordance with the Declaration of Helsinki. All patients provided written consents.

Patients with medial knee OA were selected for this analysis based on their baseline Kellgren-Lawrence (KL) scores^28^ and a 2-year follow-up. Specifically, patients were required to have a KL-score > 0 in the medial compartment and a higher KL-score in the medial compartment than in the lateral compartment at baseline. OA patients were classified as progressors if their KL score in the medial compartment increased by at least 1 point over the two-year follow up period. Patients whose KL score remained unchanged over the 2 years were categorized as non-progressors, and those with a KL score of 0 in both compartments at both time points were classified as controls. Patients with a KL-score increase of 0.5, a reduced KL score, or lateral compartment progression were excluded.

Following group allocation, a total of ten controls (C), nine progressors (P), and eleven non-progressors (NP) were retained for further analysis (Table 1). Detailed information for each subject is tabulated in supplementary Table S1. Non-parametric Kruskall-Wallis tests were performed using RStudio version 2024.09.1 + 394 (RStudio, PBC, Boston, MA) to evaluate between-group differences.

**Table 1:** Summary of the demographic characteristics of control, OA progressors, and non-progressors. KL scores for each group are detailed for the medial compartment at baseline. Detailed are average ± standard deviation for weight, height, body mass index (BMI), and age at baseline.

Group	Number of subjects	Number of subjects with different kl scores at baseline			Average weight (kg)	Average height (m)	Average BMI (kg/m2)	Average age (year)	Gait speed (m/s)	
									First peak	Second peak
		KL1	KL2	KL3						
progressor	9	6	2	1	71.7 ± 7.00	1.60 ± 0.06	28.05 ± 3.92	66.7 ± 4.1	1.24 ± 0.18	1.15 ± 0.19
non-progressor	11	7	4	0	67.1 ± 14.0	1.61 ± 0.03	25.90 ± 4.77	64.6 ± 5.6	1.19 ± 0.21	1.13 ± 0.16
control	10	-	-	-	64.2 ± 10.3	1.61 ± 0.06	24.71 ± 3.76	63.0 ± 10.2	1.20 ± 0.24	1.16 ± 0.21

Previously collected gait data were analyzed^26,27^. including marker positions tracked using a 10MX Vicon Motion capture system at 100 Hz, was synchronized with ground reaction forces acquired via AMTI in-ground force plates collected at 1000 Hz. Participants performed a static calibration trial^29^ in an anatomical position for 5 s, followed by over-ground walking trials at a self-selected pace. Marker trajectories and force plate data were processed using custom Matlab (MATLAB R2020b, The Math Works, Inc., Natick, Massachusetts, USA) scripts for subsequent musculoskeletal modeling.

### Musculoskeletal model to estimate joint contact mechanism

A state-of-the-art musculoskeletal modeling framework, OpenSim Joint Articular Mechanics (JAM) (https://github.com/clnsmith/opensim-jam),^30^ was used to estimate joint kinematics, joint moments, and contact loading parameters. First, a generic OpenSim^31^ model^32^ was scaled to match each participant’s anthropometry using static trial marker positions. OpenSim-JAM integrates a unique knee joint contact model with standard OpenSim tools to estimate force-dependent kinematics for knee joint secondary coordinates (i.e., tibiofemoral joint internal/external rotation, adduction/abduction and translations, and six-degrees-of-freedom patellofemoral joint) which are dynamically consistent with ligament, muscle, and articular contact forces. Dynamic contact pressure across the knee joint surface, including the center of pressure (COP), was estimated using an elastic foundation model formulation. Articular contact pressures and COP were separately calculated for the medial and lateral compartments.

One trial was selected randomly for each participant for further modeling. Joint angles and moments in both hip and knee joints, mean contact pressure, and center of pressure were extracted at the first and second peak of total tibiofemoral joint loading identified using a semi-automated approach through a custom-written Matlab script and exported for subsequent statistical analysis. Parameters were compared between groups at the first and second peaks of tibiofemoral joint loading. Non-parametric Kruskall-Wallis tests followed by Mann-Whitney U tests were then performed to evaluate between-group differences. All statistical analyses were performed using RStudio version 2024.09.1 + 394 (RStudio, PBC, Boston, MA).

### Finite element model to estimate cartilage mechanical response

Contact pressure estimated by MSK models was extracted for the stance phase of gait and applied to a finite element (FE) model of the medial tibial compartment, as all subjects involved in the project have medial compartment OA.

Identical generic cartilage geometries were used in the FE and MSK models. The FE model incorporated hexahedral meshes generated using ANSA (v21.0.1, BETA CAE Systems International AG, Switzerland) with 21,076 elements (C3D8P). This model used a fibril-reinforced poroelastic material (FRPE)^33–35^ formulation, which was implemented in Abaqus (Abaqus 2021, Dassault Systèmes Simulia Corp., Providence, RI, USA) via a user-defined material subroutine (UMAT). The material properties were derived from unconfined compression tests on non-OA human cartilage^36^. Detailed information on the material model and properties are documented in the supplementary material (Table S2). Subject-specific dynamic contact pressures estimated by MSK models were applied to the cartilage’s articular surface, while the cartilage’s bottom surface, attached to the subchondral bone, was fixed in the model.

Three critical mechanical responses—compressive, fibril, and maximum shear strain^14^—were analyzed at the first and second peaks of tibiofemoral joint loading and the maximum value for each element during the entire stance phase of gait. The compressive strain, representing the cartilage deformation under compressive loading, is approximated by the absolute minimum principal strain. Based on the literature and in line with our previous work, fibril strain was found indicative of fibril degradation and maximum shear strain was leading to proteoglycan depletion during OA progression^11–13,37,38^. These parameters, including minimum principal strain, fibril strain and maximum shear strain were plotted using a histogram as percentage of volume in Matlab and compared between groups.

Pilot Clustering Test to Identify OA Progressors.

Histograms of contact pressures across the medial compartment estimated by MSK models and cartilage mechanical responses estimated by FE models were used in a pilot clustering analysis to identify OA progressors using a time series k-mean clustering algorithm (tslearn^39^. Data from each subject were input into the algorithm at the first and second peaks of joint loading, and the maximum value was observed throughout the stance phase. Two clusters were generated, and each histogram was iteratively assigned to the nearest cluster centroid based on the Euclidean distance until convergency. The correctness of clustering was checked to assess the credibility using contact pressures and cartilage mechanical responses as in silico mechanical biomarkers in distinguishing progressors from non-progressors and controls.

## Results

No significant differences in BMI (*p* = 0.2878), age (*p* = 0.6707), walking speeds at the first (*p* = 0.9567) and second peaks (*p* = 0.9913) of tibiofemoral joint loading were observed.

Given the relevance of medial compartment OA progression, the current results section will specifically focus on contact mechanics and cartilage mechanical responses in medial tibial cartilage. However, for completeness, knee and hip joint angles and moments are available in supplementary material in Figures S1 and S2. In short, the progressors group showed a significantly more externally rotated knee than the control group ( *p* = 0.013) at the first peak. However, no significant differences in knee adduction moment were found between groups. Furthermore, the contact mechanism in the lateral compartment is illustrated in supplementary Figure S3.

### Joint contact mechanics

**Fig. 2:**
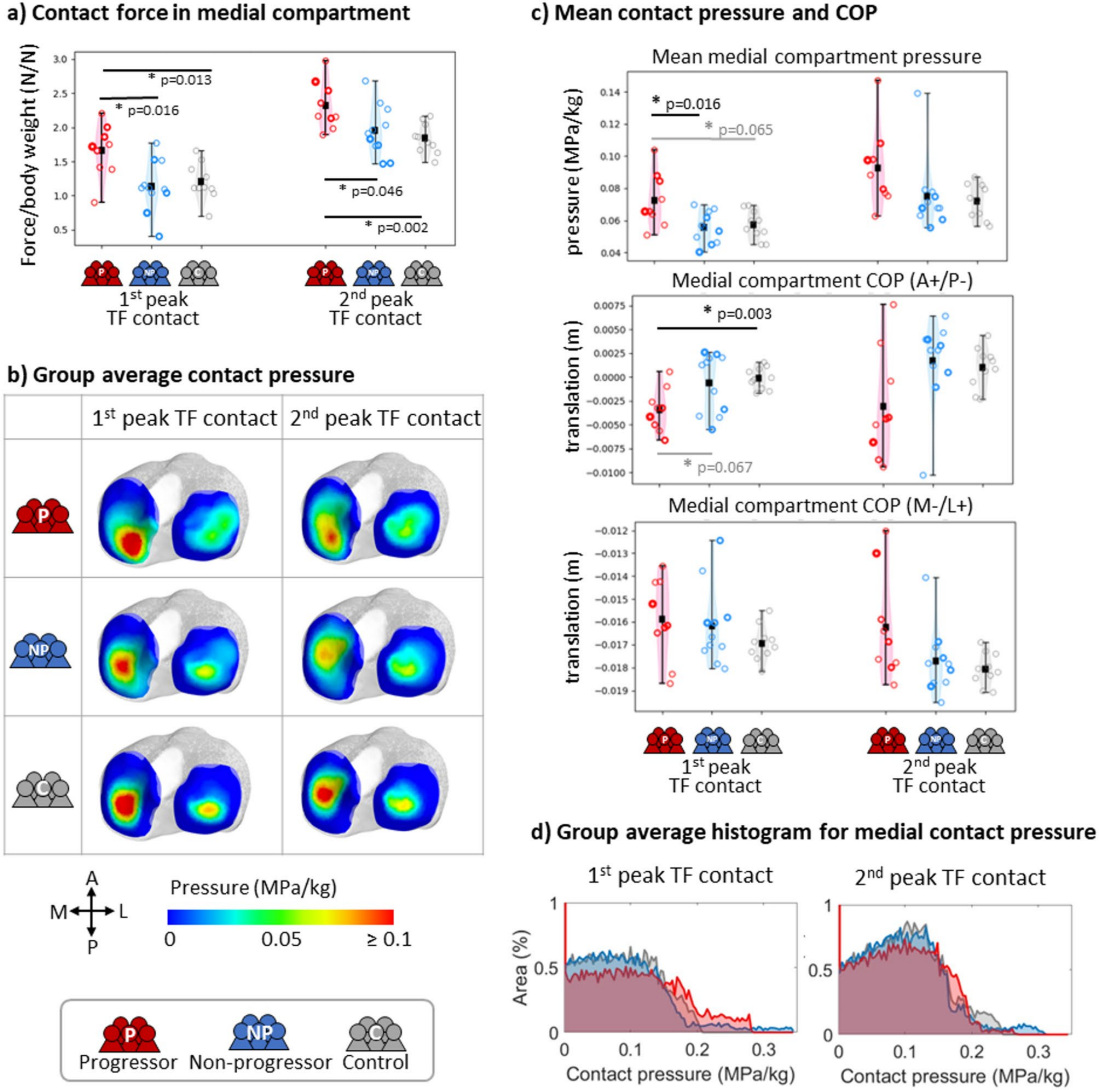
Joint contact mechanics were estimated for groups of OA progressors (red), non-progressors (blue), and control subjects (grey) at the first and second peaks of tibiofemoral (TF) contact forces. (**a**) Contact pressure in medial compartment. (**b**) Group-average tibial contact pressure maps. Anatomical directions of medial (M), lateral (L), anterior (A), and posterior (P) are noted in the bottom left corner. (**c**) Mean medial compartment cartilage contact pressure (top) and medial compartment center of pressure (COP) in the anterior/posterior (middle) and medial/lateral (bottom) direction. (**d**) Histograms of medial compartment cartilage contact pressure per area of articular contact surface for each subject group. In a) and c), individual subject values are shown by circles, with the thicker circles indicating a higher KL score at baseline. Dark squares indicate the group average and vertical lines indicate the entire range. Stars * indicate significant between-group differences (*p* < 0.05 in black and 0.05 < *p* < 0.1 in grey) using the Mann Whitney U test with a p-value marked.

Progressors exhibited significantly higher TF contact forces than both controls and non-progressors at the first and second peaks of loading (Fig. 2a). These elevated contact forces contributed to higher mean contact pressures (Fig. 2c, top) and were associated with differences in peak loading location (Fig. 2b and c, middle and bottom). In the medial compartment, the COP was more posterior in progressors than in controls and non-progressors, particularly at the first peak. At the second peak, a lateral shift of the COP was observed in progressors compared to other groups (although this did not reach statistical significance).

### Cartilage mechanical response

**Fig. 3:**
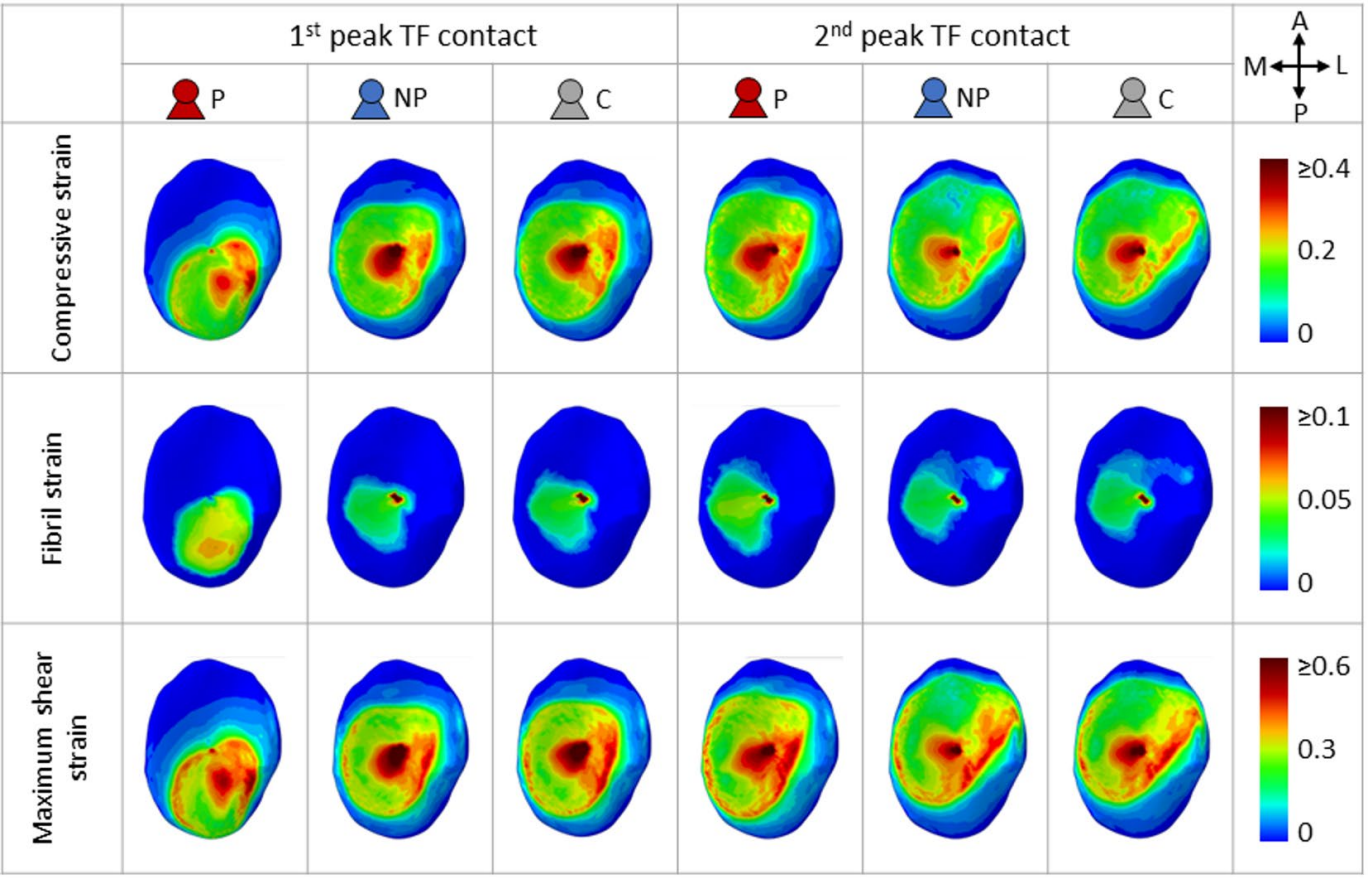
FE Simulated strain maps in the medial cartilage of compressive strain, fibril strain, and maximum shear strain of representative subjects in the subject groups of OA progressors (P09), non-progressors (NP09), and control (C08) at first and second peaks of TF contact forces. Both progressor and non-progressor score KL1 in the medial compartment at baseline. Anatomical directions of medial (M), lateral (L), anterior (A), and posterior (P) are noted in the top right corner.

Representative tibial strain maps for the medial compartment, estimated by FE models, at the first and second peaks of tibiofemoral contact force are shown in Fig. 3 for each subject group. Compressive strains were calculated using minimum principal strain in cartilage. Progressors exhibited posterolateral shifts in areas with high strain values for all studied strains at the first peak compared to non-progressors and controls. This shift mirrored the displacement of the COP in the medial compartment shown in the MSK model.

**Fig. 4:**
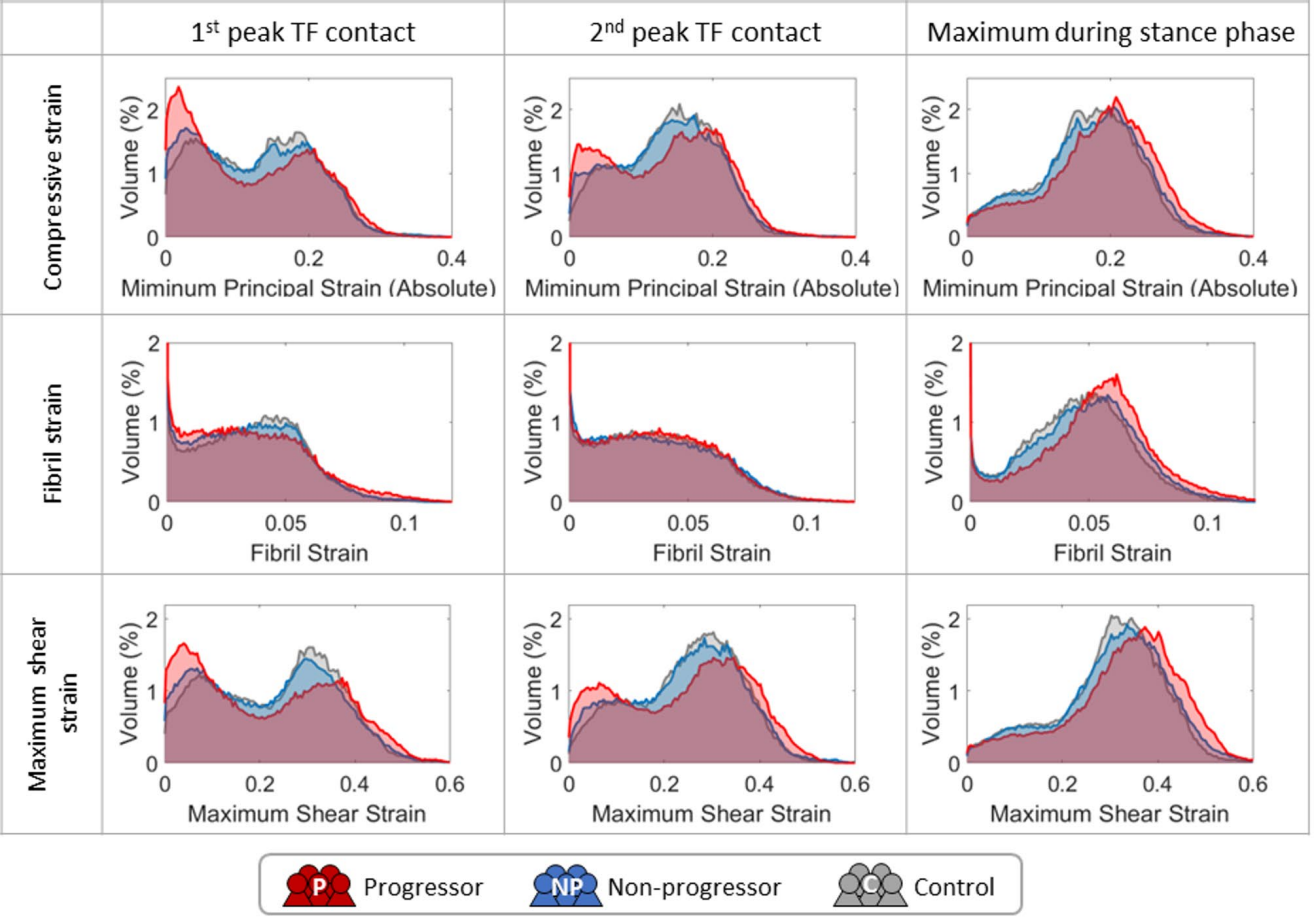
Histograms of simulated compressive strain (absolute minimum principal strain), fibril strain, and maximum shear strain per cartilage volume in cartilage medial compartment of OA progressors (red), non-progressors (blue), and controls (grey). Strains are obtained at the first (left column) and second (middle column) of TF contact force peaks. The histogram for maximum strains of each element during the stance phase is also presented in the right column.

Due to higher contact pressures, progressors showed slightly larger cartilage volume experiencing higher compressive strain, fibril strain, and maximum shear strain at the first peak compared to non-progressors and controls (Fig. 4 left). Interestingly, a larger volume of cartilage in progressors is experiencing lower strains during the first peak. Similar differences between progressors and other groups are observed at the second peak for compressive and maximum shear strain (Fig. 4 middle). However, the volume difference in cartilage under lower strains is notably less than at the first peak. For fibril strain at the second peak, no clear difference was observed between the three subject groups (Fig. 4 middle).

When examining the maximum strain of each element during the stance phase (Fig. 4 right), a clear frequency shift towards higher strains can be observed in all three mechanical responses of progressors compared to the other groups.

### Clustering analysis to identify OA progressors

**Fig. 5:**
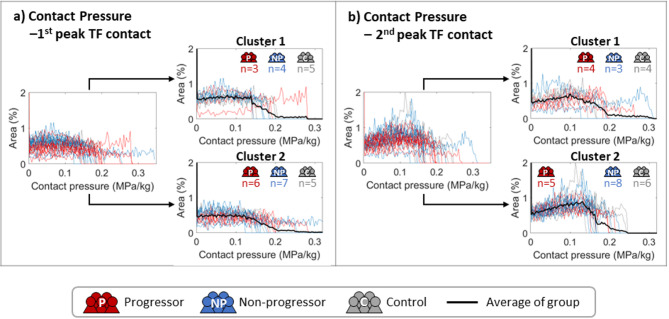
Unsupervised clustering results for contact pressure at (**a**) the first peak and (**b**) the second peak of TF contact forces. Histograms of individual OA progressors, non-progressors, and control subjects are presented in red, blue, and grey, respectively, with black lines indicating the average values of the clustered groups.

Unsupervised clustering results for contact pressures estimated from MSK modeling at the first and second peaks of contact forces are presented in Fig. 5. Detailed clustering results for each subject are listed in Supplementary Table S3.

No dominance of OA progressors (red) or non-progressors (blue) and controls (grey) was observed in either cluster.

**Fig. 6:**
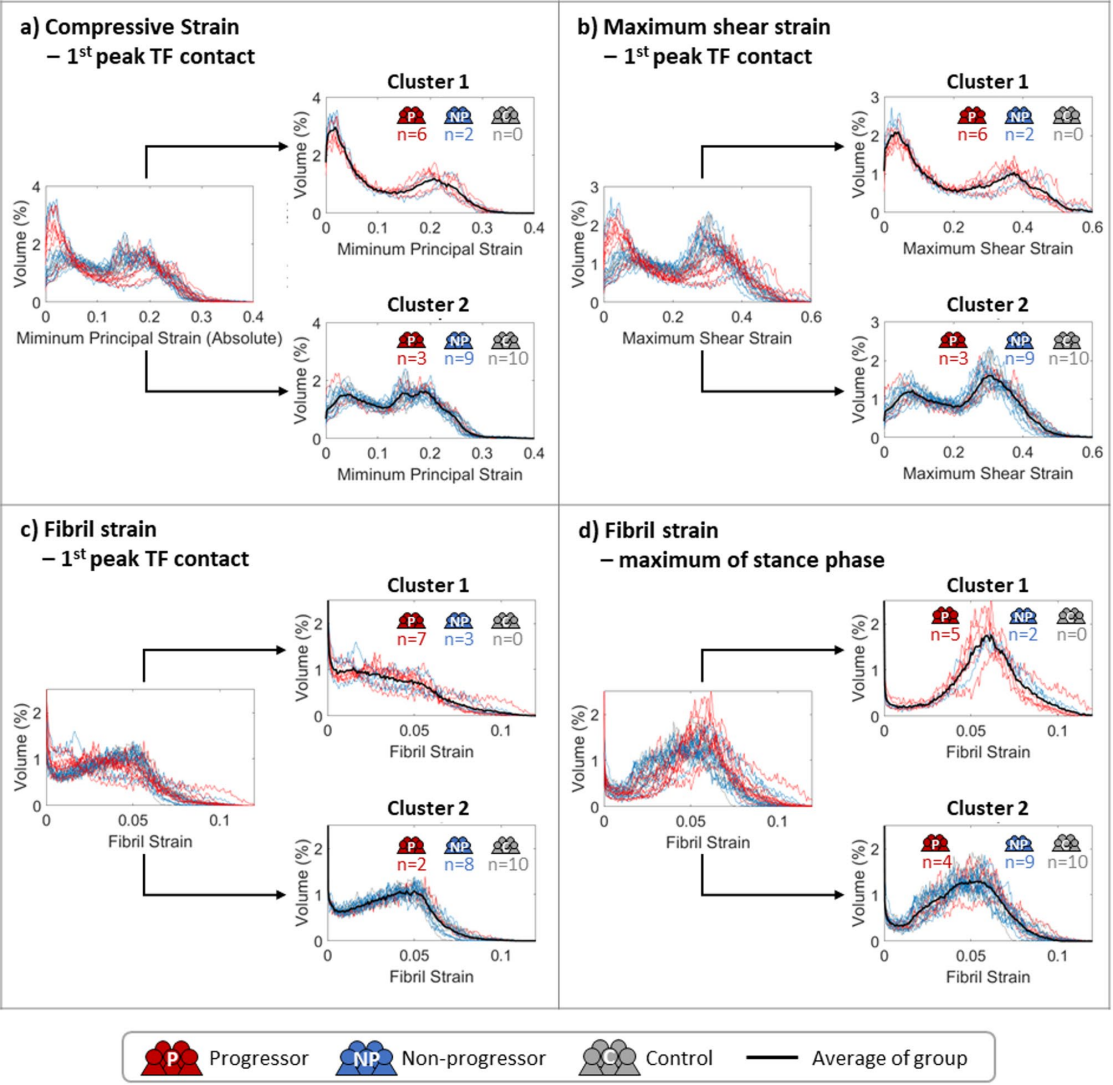
Unsupervised clustering results for histograms of key mechanical parameters, including (**a**) compressive strain (absolute minimum principal strain) at the first peak of TF contact forces, (**b**) maximum shear strain at the first peak of TF contact forces, (**c**) fibril strain at the first peak of TF contact forces, and (**d**) fibril strain at the maximum value of the stance phase. Histograms of individual OA progressors, non-progressors, and control subjects are presented in red, blue, and grey, respectively, with black lines indicating the average values of the clustered groups.

Contrary to contact pressure, cartilage mechanical responses from the FE analyses showed promising results for clustering. Unsupervised clustering results for the best-performing parameters—compressive strain and maximum shear strain both at the first peak of contact force and fibril strain at the first peak of contact force and maximum of stance phase—are shown in Fig. 6. Clustering results for other studied strains at the two peaks of contact force and maximum during the stance phase are provided in the supplementary material (Figure S4). Notably, the identified cluster 1 is dominated by OA progressors (in red), while cluster 2 is dominated by non-progressors (in blue) and control subjects (in grey). Compressive and maximum shear strains at the first peak of tibiofemoral joint contact forces achieved identical clustering performance, with Cluster 1 comprising 66.7% of progressors and 18.2% of non-progressors (false positive). Cluster 2 contained 33.3% of progressors (false negative), 81.1% of non-progressors, and all controls.

In comparison, fibril strain at the first peak showed more false positives (27.3% of non-progressors in Cluster 1) but fewer false negatives (22.2% of progressors in Cluster 2). At the maximum value during the stance phase, fibril strain classified more false negatives (44.4% of progressors in Cluster 2), though some of these misclassifications did not overlap with those identified by the compressive strain and maximum shear strain at the first peak.

## Discussion

This study is the first to identify mechanical cartilage response during gait as biomechanical biomarkers that can distinguish patients with progressing medial knee OA from those with non-progressing OA or controls based on patient-specific gait data.

This longitudinal study confirms increased contact pressures and a posterolateral shift of COP (Fig. 2) in the medial compartment were observed in patients with accelerated OA progression. This increase in loading magnitude and changes in loading areas among progressors supports the hypothesis that in OA, cartilage mechanical environment is altered and make therefore disrupted the homeostasis due to an imbalance between joint loading and load-bearing capacity^40^. Our findings align with previous literature, which reported a more posterolateral COP in patients with established OA than controls^16^. The altered loading patterns suggest that changes in the location of the loading, rather than just the magnitude, may drive OA progression in its early stages.

Although the MSK model employs an elastic foundation to predict cartilage contact pressures, its load-dependant behaviour has been verified using dynamic MRI measures^32^. Moreover, the predicted contact locations closely match those estimated by the whole joint FE model using fibril-reinforced material previously developed in our group^14^. The magnitude of predicted pressure also falls within the range of published results of whole joint FE models^14,41–43^; importantly, the relative increase observed in OA progressors compared to controls is consistent with earlier findings^14,41^ thereby supporting reliable inter-subject and inter-group comparisons.

The finding that no significant differences were found in the knee adduction moments (KAM) of progressors compared to non-progressors or controls further highlights their utility and the need to consider direct estimates of joint loading – rather than analyzing joint level kinematics and moments (supplementary Figure S1). This contrasts previous literature suggesting KAM may act as a mediator for medial knee OA^44,45^. Within this study and others, the KAM’s ability as a direct surrogate for joint contact forces is becoming increasingly unclear.

Although MSK-estimated joint contact mechanics showed statistically significant differences between progressors and non-progressors or controls, considerable overlaps across groups limited discriminative power.

To identify more specific in silico biomarkers, we investigated cartilage tissue responses using FRPE FE models. Fibril strain and maximum shear strain, which have been associated with fibril degradation and proteoglycan depletion^11,13,14,46^ respectively, provided stronger discriminatory value. Notably, a larger cartilage volume in progressors exceeded established degeneration thresholds for collagen and proteoglycan depletion derived from in vitro studies (fibril strain = 0.1^13,14^ and maximum shear strain = 0.4^46^ or 0.5^11^, respectively), aligning with previous computational work^47,48^ but for the first time, distinguishing OA progressors from non-progressors.

At the first and second peaks of tibiofemoral (TF) contact forces, cartilage mechanical response does not always increase proportionally as contact loading increases due to cartilage’s non-linear and time-dependent nature. Signature histograms of compressive and maximum shear strains revealed a dual trend in progressors, where a larger volume of elements experienced low-range and high-range strains at the first and second peaks. This dual pattern indicated OA progressors experiencing both under- and overloading at the first and second peaks, suggesting that an altered loading pattern, rather than just increased magnitude, could also discriminate OA progressors.

Interestingly, neither the contact mechanics nor cartilage mechanical responses differed between non-progressors and controls. Although earlier studies reported elevated knee joint loading^16,17,49^ and increased cartilage volumes exceeding degeneration thresholds^47,48^ in patients with knee OA compared to controls, these did not distinguish between progressing and non-progressing OA patients. Therefore, previously reported differences observed between OA patients and controls align primarily with progressors within the OA group.

The unsupervised clustering presented here should be considered exploratory, as the small sample size limits the robustness of classification. Nevertheless, by operating without prior knowledge of subject groups, the analysis captured characteristic strain signatures and provided a proof-of-concept that FE-derived cartilage responses may serve as sensitive biomarkers of OA progression. However, larger longitudinal datasets will be required to validate and refine these biomarkers for clinical application.

Indeed, investigating more in detail individual subject results, two progressors and two non-progressors exhibited deviant mechanical responses that resembled the opposite group’s characteristics (see supplementary Table S3, P01, P07, NP07 and NP08). These four subjects accounted for most of the false clustering results. Moreover, no specific characteristics were found in joint kinematics or age of those 4 outliners. However, the progressor outliners (P01 and P07) showed higher BMI compared to the group average (Tables S1 and 1), indicating high joint loading. Whereas, the non-progressor outliners (NP07 and NP08) had lower BMI compared to their group average (Tables S1 and 1). Furthermore, it is essential to acknowledge that mechanical loading and joint kinematics during gait are not the only factors contributing to cartilage degeneration during OA progression. Incorporating other factors, such as systemic inflammation, genetic predisposition, and high-impact physical activities^50,51^ could provide a more comprehensive understanding of OA structural progression and thus improve prediction accuracy for future clinical applications.

While this study offers valuable insights, certain aspects could be refined to enhance the prediction of OA progression in future investigations. For instance, the study relied on clinical standard KL-score to assess structural OA progression. However, the assessment is based on x-ray imaging, which lacks the ability to capture detailed cartilage degeneration in the joint Future studies could incorporate Magnetic Resonance Imaging (MRI) to examine cartilage degeneration more precisely and compare the degeneration location with high-strain regions estimated by the in silico model, further validating the model prediction. In terms of the modelling framework, although the cartilage geometry was scaled to the size of each patient in the models, patient-specific cartilage geometry and tibiofemoral alignment were not included in the current workflow. These anatomical variations could further alter the contact mechanism in the joint^52^ potentially changing the mechanical responses of the cartilage. To incorporate patient-specific anatomy, a recent MSK model that directly estimates joint contact mechanics, including patient-specific cartilage geometry and tibiofemoral alignment^53^ could be integrated within the current MSK-FE modeling workflow as a next step. Additionally, this study used identical cartilage material properties for both control and OA subjects. This choice seems valid as most OA subjects in this study had a KL score of 1 or 2 at baseline, indicating early or moderate OA with limited cartilage degeneration^54^. However, incorporating OA state-specific material properties and initial cartilage conditions in future studies could enhance the workflow’s ability to predict in vivo cartilage degeneration. Lastly, the study focused on the chronic mechanical alterations caused by gait patterns in OA progressors. Post-traumatic OA progression, which can result from ligament rupture, cartilage lesions, or meniscus tears, is not assessed. As post-traumatic OA progression is particularly relevant for younger patients, our relevance of this workflow adapted to include the effect of meniscal injuries, cartilage lesions, or changes in ligament properties needs to be analysed in future studies.

While high personalization of joint geometry and cartilage mechanical properties could potentially enhance the accuracy of estimated cartilage mechanical responses, achieving this would require MRI-based acquisition, substantially raising clinical costs and computational demands. Although such personalization may eventually benefit patient-tailored surgical and rehabilitation programs, it would limit the feasibility of large-scale joint health screening of older people.

Joint health screening would enable early identification of individuals at risk of fast OA progression, allowing for timely preventive interventions to slow disease progression and preserve joint function. To make such “early warning” screenings viable at a population level, cost-effectiveness and prediction accuracy must be balanced. By relying on gait-derived loading parameters with generic but scaled geometries, our workflow balances efficiency with predictive accuracy, enabling the identification for OA progressors in a manner that is cost-effective and scalable. Compared to previous MSK-FE workflows^14,24,47^ our approach reduces computational cost by a factor of 8, minimizes model complexity and avoids convergence challenges, thereby strengthening its potential for clinical application in early risk screening.

In addition to the cost-effective modelling workflow for deriving mechanical biomarkers, efficient gait collection should be employed for future clinical applications. Recent developments in markerless motion capture^55^ and portable devices (e.g. inertial measurement unit (IMUs))^56^ have shown potential to replicate motions and reaction forces typically captured in laboratory settings using high-speed cameras with markers and force plates. If proofed sufficiently accurate in an OA context, these technologies would enable rapid and low-burden gait data collection and assessments in clinical environments, providing essential input for calculating mechanical biomarkers and estimating OA progression risk.

Validation is essential before translating the proposed workflow into clinical applications. However, direct in vivo measurements of joint contact pressure or cartilage strains are not feasible due to its invasive nature. Although previous studies attempted to validate estimated contact pressures or forces against cadaveric experiments^57,58^ or instrumented prosthetic data^59^ these approaches have inherent limitations, including impaired muscle function, ligaments integrity and altered joint loading and alignment, which could not fully represent in vivo joint mechanics. Quantitative MRi (qMRi) enable cartilage constituents assessments such as collagen integrity, proteoglycan and water content^60^. This provides direct validation of the proposed mechanical biomarkers - fibril stain and maximum shear strain - which indicate degenerations of collagen and proteoglycan, respectively^37^. Future work will use qMRI to validate the spatial correspondence between elevated mechanical biomarkers and cartilage degeneration in a newly recruited cohort, thereby enabling subject-specific validation of the modelling framework.

In conclusion, using an integrated MSK-FE modeling approach, cartilage mechanical responses during gait effectively distinguished medial knee OA progressors (over two years) from non-progressors and controls. These mechanical responses could serve as sensitive in silico biomarkers to identify patients at risk of accelerated OA progression, further supported by a pilot unsupervised clustering analysis. With future validation in larger cohorts, this workflow could be adapted for clinical application, enabling screening for joint health in the elderly and early identification of patients at risk of OA progression. This would allow timely access to preventive interventions such as weight management, neuromuscular exercise^61^ and gait retraining^62^helping to preserve joint function and mobility and reduce risks of mobility loss.

## Supplementary Information

Below is the link to the electronic supplementary material.

Supplementary Material 1

## Data Availability

The datasets generated during and/or analysed during the current study are available from the corresponding author on request.

## References

[1] Steinmetz, J. D. et al. Global, regional, and National burden of osteoarthritis, 1990–2020 and projections to 2050: a systematic analysis for the global burden of disease study 2021. *Lancet Rheumatol.* **5**, e508–e522 (2023). 37675071 10.1016/S2665-9913(23)00163-7 PMC10477960

[2] Hunter, D. J., Schofield, D. & Callander, E. The individual and socioeconomic impact of osteoarthritis. *Nature Reviews Rheumatology* vol. 10 437–441 at (2014). 10.1038/nrrheum.2014.44 10.1038/nrrheum.2014.44 24662640

[3] Lories, R. J. & Luyten, F. P. The bone-cartilage unit in osteoarthritis. *Nat. Rev. Rheumatol.* **7**, 43–49 (2011). 21135881 10.1038/nrrheum.2010.197

[4] Conley, B. et al. Core recommendations for osteoarthritis care: A systematic review of clinical practice guidelines. *Arthritis Care Res.* **75**, 1897–1907 (2023). 10.1002/acr.25101 PMC10952362 36762545

[5] Marriott, K. A. & Birmingham, T. B. Fundamentals of osteoarthritis. Rehabilitation: exercise, diet, biomechanics, and physical therapist-delivered interventions. *Osteoarthr. Cartil.* **31**, 1312–1326 (2023). 10.1016/j.joca.2023.06.011 37423596

[6] Emrani, P. S. et al. Joint space narrowing and Kellgren-Lawrence progression in knee osteoarthritis: an analytic literature synthesis. *Osteoarthr. Cartil.* **16**, 873–882 (2008). 10.1016/j.joca.2007.12.004 PMC2701468 18280757

[7] Weinstein, A. M. et al. Estimating the burden of total knee replacement in the united States. *J. Bone Jt. Surg.* **95**, 385–392 (2013). 10.2106/JBJS.L.00206 PMC3748969 23344005

[8] Bader, D. L., Salter, D. M. & Chowdhury, T. T. Biomechanical influence of Cartilage Homeostasis in Health and Disease. *Arthritis* 1–16 (2011). (2011). 10.1155/2011/979032 PMC3196252 22046527

[9] Sun, H. B. Mechanical loading, cartilage degradation, and arthritis. *Ann. N Y Acad. Sci.* **1211**, 37–50 (2010). 21062294 10.1111/j.1749-6632.2010.05808.x

[10] Varady, N. H. & Grodzinsky, A. J. Osteoarthritis year in review 2015: mechanics. *Osteoarthr. Cartil.* **24**, 27–35 (2016). 10.1016/j.joca.2015.08.018 PMC4693146 26707990

[11] Orozco, G. A., Tanska, P., Florea, C., Grodzinsky, A. J. & Korhonen, R. K. A novel Mechanobiological model can predict how physiologically relevant dynamic loading causes proteoglycan loss in mechanically injured articular cartilage. *Sci. Rep.* **8**, 1–16 (2018). 30348953 10.1038/s41598-018-33759-3 PMC6197240

[12] Eskelinen, A. S. A., Mononen, M. E., Venäläinen, M. S., Korhonen, R. K. & Tanska, P. Maximum shear strain-based algorithm can predict proteoglycan loss in damaged articular cartilage. *Biomech. Model. Mechanobiol.* **18**, 753–778 (2019). 30631999 10.1007/s10237-018-01113-1

[13] Elahi, S. A. et al. An in Silico framework of cartilage degeneration that integrates fibril reorientation and degradation along with altered hydration and fixed charge density loss. *Front Bioeng. Biotechnol* **9**, (2021). 10.3389/fbioe.2021.680257 PMC8258121 34239859

[14] Mohout, I. et al. Signatures of disease progression in knee osteoarthritis: insights from an integrated multi-scale modeling approach, a proof of concept. *Front. Bioeng. Biotechnol.* **11**, 1–12 (2023). 10.3389/fbioe.2023.1214693 PMC10413555 37576991

[15] Smith, C. R., Choi, W., Negrut, K., Thelen, D. G. & D. & Efficient computation of cartilage contact pressures within dynamic simulations of movement. *Comput. Methods Biomech. Biomed. Eng. Imaging Vis.* **6**, 491–498 (2018). 30740280 10.1080/21681163.2016.1172346 PMC6366837

[16] Meireles, S. et al. Medial knee loading is altered in subjects with early osteoarthritis during gait but not during step-up-and-over task. *PLoS One* **12**, (2017). 10.1371/journal.pone.0187583 PMC5678707 29117248

[17] Marouane, H., Shirazi-Adl, A. & Adouni, M. Alterations in knee contact forces and centers in stance phase of gait: A detailed lower extremity musculoskeletal model. *J. Biomech.* **49**, 185–192 (2016). 26708962 10.1016/j.jbiomech.2015.12.016

[18] Kumar, R. et al. Polarization second harmonic generation microscopy provides quantitative enhanced molecular specificity for tissue diagnostics. *J. Biophotonics*. **8**, 730–739 (2015). 25363416 10.1002/jbio.201400086

[19] Adouni, M. & Shirazi-Adl, A. Evaluation of knee joint muscle forces and tissue stresses-strains during gait in severe OA versus normal subjects. *J. Orthop. Res.* **32**, 69–78 (2014). 24038150 10.1002/jor.22472

[20] Halonen, K. S., Mononen, M. E., Jurvelin, J. S., Töyräs, J. & Korhonen, R. K. Importance of depth-wise distribution of collagen and proteoglycans in articular cartilage-A 3D finite element study of stresses and strains in human knee joint. *J. Biomech.* **46**, 1184–1192 (2013). 23384762 10.1016/j.jbiomech.2012.12.025

[21] Halonen, K. S. et al. Deformation of articular cartilage during static loading of a knee joint - Experimental and finite element analysis. *J. Biomech.* **47**, 2467–2474 (2014). 24813824 10.1016/j.jbiomech.2014.04.013

[22] Halonen, K. S. et al. Importance of patella, quadriceps forces, and depthwise cartilage structure on knee joint motion and cartilage response during gait. *J. Biomech. Eng.* **138**, 1–11 (2016). 10.1115/1.4033516 27138135

[23] Erdemir, A. et al. Deciphering the ‘art’ in modeling and simulation of the knee joint: overall strategy. *J. Biomech. Eng.* **141**, 1–10 (2019). 10.1115/1.4043346 PMC6611350 31166589

[24] Esrafilian, A. et al. EMG-Assisted muscle force driven finite element model of the knee joint with Fibril-Reinforced poroelastic cartilages and menisci. *Sci. Rep.* **10**, 1–16 (2020). 32080233 10.1038/s41598-020-59602-2 PMC7033219

[25] Orozco, G. A. et al. Shear strain and inflammation-induced fixed charge density loss in the knee joint cartilage following ACL injury and reconstruction: A computational study. *J. Orthop. Res.* **40**, 1505–1522 (2022). 34533840 10.1002/jor.25177 PMC8926939

[26] Baert, I. A. C. et al. Gait characteristics and lower limb muscle strength in women with early and established knee osteoarthritis. *Clin. Biomech.* **28**, 40–47 (2013). 10.1016/j.clinbiomech.2012.10.007 23159192

[27] Mahmoudian, A. et al. Varus thrust in women with early medial knee osteoarthritis and its relation with the external knee adduction moment. *Clin. Biomech.* **39**, 109–114 (2016). 10.1016/j.clinbiomech.2016.10.006 27744006

[28] Kellgren, J. H. Radiological assessment. *Ann. Rheum. Dis.* 494–502. 10.2307/3578513 (1957). 10.1136/ard.16.4.494 PMC1006995 13498604

[29] Cappozzo, Catani, F. & Della Croce, U. Leardini, a. Position and Orietnation in space of bones during movement. *Clin. Biomech.* **10**, 171–178 (1995). 10.1016/0268-0033(95)91394-t 11415549

[30] Smith, C. R., Lenhart, R. L., Kaiser, J., Vignos, M. F. & Thelen, D. G. Influence of ligament properties on tibiofemoral mechanics in walking. *J. Knee Surg.* **29**, 99–106 (2014). 10.1055/s-0035-1558858 PMC4755512 26408997

[31] Delp, S. L. et al. OpenSim: Open-source software to create and analyze dynamic simulations of movement. *IEEE Trans. Biomed. Eng.* **54**, 1940–1950 (2007). 18018689 10.1109/TBME.2007.901024

[32] Lenhart, R. L., Kaiser, J., Smith, C. R. & Thelen, D. G. Prediction and validation of Load-Dependent behavior of the tibiofemoral and patellofemoral joints during movement. *Ann. Biomed. Eng.* **43**, 2675–2685 (2015). 25917122 10.1007/s10439-015-1326-3 PMC4886716

[33] Ebrahimi, M. et al. Elastic, viscoelastic and Fibril-Reinforced poroelastic material properties of healthy and Osteoarthritic human tibial cartilage. *Ann. Biomed. Eng.* **47**, 953–966 (2019). 30690688 10.1007/s10439-019-02213-4 PMC8494710

[34] Ebrahimi, M. et al. Structure–Function relationships of healthy and Osteoarthritic human tibial cartilage: experimental and numerical investigation. *Ann. Biomed. Eng.* **48**, 2887–2900 (2020). 32648191 10.1007/s10439-020-02559-0 PMC7723942

[35] Elahi, S. A. et al. Guide to mechanical characterization of articular cartilage and hydrogel constructs based on a systematic in Silico parameter sensitivity analysis. *J Mech. Behav. Biomed. Mater* **124**, (2021). 10.1016/j.jmbbm.2021.104795 34488174

[36] Castro-Vinuelas, S. A. E. R. & Govaerts, A. Unconfined compression experimental protocol for cartilage explants and hydrogel constructs: from sample Preparation to mechanical characterization. *Cartil. Tissue Engineering: Introduction*. 271–287. 10.1007/978-1-0716-2839-3_1 (2023). 10.1007/978-1-0716-2839-3_19 36355298

[37] Elahi, S. A. et al. Contribution of collagen degradation and proteoglycan depletion to cartilage degeneration in primary and secondary osteoarthritis: an in Silico study. *Osteoarthr. Cartil.* **31**, 741–752 (2023). 10.1016/j.joca.2023.01.004 36669584

[38] Hosseini, S. M., Wilson, W., Ito, K. & Van Donkelaar, C. C. A numerical model to study mechanically induced initiation and progression of damage in articular cartilage. *Osteoarthr. Cartil.* **22**, 95–103 (2014). 10.1016/j.joca.2013.10.010 24185112

[39] Tavenard, R. et al. A machine learning toolkit for time series data. *J. Mach. Learn. Res.* **21**, 1–6 (2020). Tslearn.34305477

[40] Andriacchi, T. P., Smith, R. L., Medicine, S. & Koo, S. A framework for the in vivo pathomechanics of osteoarthritis at the knee: 2nd special edition on musculoskeletal bioengineering. Guest editor : Kyriacos A. A framework for the in vivo pathomechanics of osteoarthritis at the knee. (2004). 10.1023/b:abme.0000017541.82498.37 15095819

[41] Venäläinen, M. S. et al. Quantitative evaluation of the mechanical risks caused by focal cartilage defects in the knee. *Sci Rep* **6**, (2016). 10.1038/srep37538 PMC5126640 27897156

[42] Halonen, K. S. et al. Workflow assessing the effect of gait alterations on stresses in the medial tibial cartilage - Combined musculoskeletal modelling and finite element analysis. *Sci. Rep.* **7**, 1–14 (2017). 29234021 10.1038/s41598-017-17228-x PMC5727195

[43] Karimi Dastgerdi, A. et al. Validation and evaluation of subject-specific finite element models of the pediatric knee. *Sci. Rep.* **13**, 1–13 (2023). 37884632 10.1038/s41598-023-45408-5 PMC10603053

[44] Miyazaki, T. et al. Dynamic load at baseline can predict radiographic disease progression in medial compartment knee osteoarthritis. *Ann. Rheum. Dis.* **61**, 617–622 (2002). 12079903 10.1136/ard.61.7.617 PMC1754164

[45] Bennell, K. L. et al. Higher dynamic medial knee load predicts greater cartilage loss over 12 months in medial knee osteoarthritis. *Ann. Rheum. Dis.* **70**, 1770–1774 (2011). 21742637 10.1136/ard.2010.147082

[46] Orozco, G. A. et al. Prediction of local fixed charge density loss in cartilage following ACL injury and reconstruction: A computational proof-of-concept study with MRI follow-up. *J. Orthop. Res.* **39**, 1064–1081 (2021). 32639603 10.1002/jor.24797 PMC7790898

[47] Liukkonen, M. K. et al. Simulation of subject-specific progression of knee osteoarthritis and comparison to experimental follow-up data: data from the osteoarthritis initiative. *Sci. Rep.* **7**, 1–14 (2017). 28835668 10.1038/s41598-017-09013-7 PMC5569023

[48] Orozco, G. A. et al. Effect of patient specificity on predicting knee cartilage degeneration in obese adults: musculoskeletal finite-element modeling of data from the CAROT trial. *J. Orthop. Res.*10.1002/jor.25912 (2024). 39031826 10.1002/jor.25912

[49] Kumar, D., Manal, K. T. & Rudolph, K. S. Knee joint loading during gait in healthy controls and individuals with knee osteoarthritis. *Osteoarthr. Cartil.* **21**, 298–305 (2013). 10.1016/j.joca.2012.11.008 PMC3804122 23182814

[50] Greene, M. A. & Loeser, R. F. Aging-related inflammation in osteoarthritis. *Osteoarthr. Cartil.* **23**, 1966–1971 (2015). 10.1016/j.joca.2015.01.008 PMC4630808 26521742

[51] Martel-Pelletier, J. et al. Osteoarthritis. *Nature Reviews Disease Primers* vol. 2 at (2016). 10.1038/nrdp.2016.72 10.1038/nrdp.2016.72 27734845

[52] Willems, M. et al. Population-based in Silico modeling of anatomical shape variation of the knee and its impact on joint loading in knee osteoarthritis. *J. Orthop. Res.* **1–12**10.1002/jor.25934 (2024). 10.1002/jor.25934 39096157

[53] Killen, B. A., Willems, M. & Jonkers, I. P. R. E. P. R. I. N. T. An Open-source framework for the generation of opensim models with personalised knee joint geometries for the Estimation of articular contact mechanics. *J. Biomech.* **177**, 112387 (2024). 39488193 10.1016/j.jbiomech.2024.112387

[54] Roemer, F. W. et al. Heterogeneity of cartilage damage in Kellgren and Lawrence grade 2 and 3 knees: the MOST study. *Osteoarthr. Cartil.* **30**, 714–723 (2022). 10.1016/j.joca.2022.02.614 PMC9433455 35202808

[55] Das, K., de Paula Oliveira, T. & Newell, J. Comparison of markerless and marker-based motion capture systems using 95% functional limits of agreement in a linear mixed-effects modelling framework. *Sci. Rep.* **13**, 1–15 (2023). 38129434 10.1038/s41598-023-49360-2 PMC10739832

[56] Raimondo, G. et al. Peak tibiofemoral contact forces estimated using IMU-Based approaches are not significantly different from motion Capture-Based estimations in patients with knee osteoarthritis. (2023). 10.3390/s23094484 PMC10181595 37177688

[57] Gu, W. & Pandy, M. G. Direct validation of human knee-joint contact mechanics derived from subject-specific finite-element models of the tibiofemoral and patellofemoral joints. *J. Biomech. Eng.* **142**, 1–10 (2020). 10.1115/1.4045594 31802099

[58] Chastain, K. et al. ACL transection results in a posterior shift and increased velocity of contact on the medial tibial plateau. *J. Biomech.* **144**, 111335 (2022). 36252309 10.1016/j.jbiomech.2022.111335

[59] Bergmann, G. et al. Standardized loads acting in knee implants. *PLoS One* **9**, (2014). 10.1371/journal.pone.0086035 PMC3900456 24465856

[60] Linka, K. et al. Machine learning-augmented and microspectroscopy-informed multiparametric MRI for the non-invasive prediction of articular cartilage composition osteoarthritis. *Osteoarthr. Cartil.* **29**, 592–602 (2021). 10.1016/j.joca.2020.12.022 33545330

[61] Roos, E. M. & Arden, N. K. Strategies for the prevention of knee osteoarthritis. *Nat. Rev. Rheumatol.* **12**, 92–101 (2016). 26439406 10.1038/nrrheum.2015.135

[62] Richards, R., van den Noort, J. C., Dekker, J. & Harlaar, J. Gait retraining with Real-Time biofeedback to reduce knee adduction moment: systematic review of effects and methods used. *Arch. Phys. Med. Rehabil*. **98**, 137–150 (2017). 27485366 10.1016/j.apmr.2016.07.006

