# Supplementary material for: Cartilage mechanical responses during gait as in silico biomarkers for medial knee OA progression
Source: Sci Rep. 2025 Oct 9;15:35303. doi: 10.1038/s41598-025-19371-2 (PMC12511356; doi:10.1038/s41598-025-19371-2)
Supplement: Supplementary file 1 — Supplementary Material 1 [file 41598_2025_19371_MOESM1_ESM.docx]

Cartilage mechanical responses during gait as *in silico* biomarkers for medial knee OA progression

Yixuan Zhang^1,+,*^, Bryce A Killen^1,+^, Ikram Mohout^1^, Miel Willems^1^, Frank P Luyten^2^, Sabine Verschueren^3^, Seyed Ali Elahi^1, 4,^ *^†^* , Ilse Jonkers^1,^ *^†^*

^1^Department of Movement Science, KU Leuven, Leuven, Belgium

^2^Skeletal Biology and Engineering Research Center, Department of Development and Regeneration, KU Leuven, Belgium

^3^Department of Rehabilitation Science, KU Leuven, Belgium

^4^Division of Biomechanics, Department of Mechanical Engineering, KU Leuven, Leuven, Belgium

^*^Corresponding [yixuan.zhang@kuleuven.be](mailto:yixuan.zhang@kuleuven.be)

^+,^ *^†^*These authors contributed equally to this work

## Demographic characteristics of individual subjects

**Table S1.** Demographic characteristics of individual control (grey), OA progressors (red) and non-progressors (blue). For control and OA non-progressors, KL scores are listed for medial and lateral compartments on both sides at baseline. For OA progressors, KL scores at both baseline and two-year follow-up are listed, if changes occurred in the specific compartment. * marked the selected compartment for finite element modelling.

| **Subject** | | **Weight (kg)** | **Height (m)** | **BMI (kg/m^2^)** | **Age (year)** | **Gait Speed (m/s)** | | **KL score** | | | |
| --- | --- | --- | --- | --- | --- | --- | --- | --- | --- | --- | --- |
|  |  |  |  |  |  | **First peak** | **Second peak** | **Left medial** | **Left lateral** | **Right medial** | **Right lateral** |
| **Control** | **C01** | 60.6 | 1.64 | 22.50 | 78 | 1.12 | 1.06 | 0 | 0 | 0* | 0 |
|  | **C02** | 54.3 | 1.5 | 24.10 | 57 | 1.46 | 1.18 | 0 | 0 | 0* | 0 |
|  | **C03** | 56.7 | 1.65 | 20.80 | 67 | 0.79 | 0.85 | 0 | 0 | 0* | 0 |
|  | **C04** | 80.2 | 1.63 | 30.18 | 63 | 1.24 | 1.10 | 0 | 0 | 0* | 0 |
|  | **C05** | 67.3 | 1.6 | 26.29 | 63 | 1.03 | 0.93 | 0* | 0 | 0 | 0 |
|  | **C06** | 84.7 | 1.61 | 32.68 | 68 | 1.23 | 1.22 | 0 | 0 | 0* | 0 |
|  | **C07** | 60.5 | 1.56 | 24.86 | 70 | 1.54 | 1.44 | 0 | 0 | 0* | 0 |
|  | **C08** | 52.6 | 1.56 | 21.61 | 62 | 0.94 | 0.94 | 0 | 0 | 0* | 0 |
|  | **C09** | 67.7 | 1.72 | 22.88 | 37 | 1.56 | 1.50 | 0* | 0 | 0 | 0 |
|  | **C10** | 57.1 | 1.64 | 21.23 | 65 | 1.39 | 1.39 | 0* | 0 | 0 | 0 |
| **Progressors** | **P01** | 81.6 | 1.59 | 32.30 | 66 | 1.15 | 0.99 | 1*-2 | 2 | 2 | 1 |
|  | **P02** | 75.6 | 1.65 | 27.80 | 69 | 1.10 | 1.08 | 2*-3 | 2 | 1 | 1 |
|  | **P03** | 59.8 | 1.7 | 20.69 | 59 | 1.65 | 1.60 | 2*-3 | 0 | 1 | 0 |
|  | **P04** | 77.7 | 1.53 | 33.19 | 65 | 1.30 | 1.19 | 1-2 | 0 | 1*-2 | 0 |
|  | **P05** | 66.4 | 1.63 | 24.99 | 63 | 1.37 | 1.27 | 0 | 0 | 3*-4 | 1 |
|  | **P06** | 79.8 | 1.6 | 31.17 | 65 | 1.08 | 1.03 | 1*-2 | 1 | 1 | 1 |
|  | **P07** | 68.9 | 1.56 | 28.31 | 72 | 1.25 | 1.12 | 1*-2 | 1 | 1 | 1 |
|  | **P08** | 64.8 | 1.65 | 24 | 73 | 1.22 | 1.14 | 1-2 | 1 | 1*-2 | 4 |
|  | **P09** | 70.6 | 1.53 | 30 | 68 | 1.02 | 0.92 | 1 | 0 | 1*-2 | 0 |
| **Non-progressors** | **NP01** | 95.6 | 1.68 | 33.87 | 58 | 1.51 | 1.38 | 1 | 0 | 2* | 0 |
|  | **NP02** | 45 | 1.55 | 18.70 | 68 | 1.27 | 0.97 | 0 | 0 | 2* | 1 |
|  | **NP03** | 65.9 | 1.64 | 24.50 | 70 | 1.16 | 1.18 | 1 | 0 | 1* | 0 |
|  | **NP04** | 81.5 | 1.57 | 33.06 | 73 | 1.23 | 1.12 | 1 | 0 | 2* | 0 |
|  | **NP05** | 52.6 | 1.58 | 21.07 | 62 | 1.27 | 1.06 | 1* | 0 | 0 | 0 |
|  | **NP06** | 62.5 | 1.61 | 24.11 | 69 | 0.77 | 0.85 | 2 | 1 | 2* | 1 |
|  | **NP07** | 60.5 | 1.6 | 23.63 | 66 | 0.93 | 0.99 | 1 | 0 | 1* | 0 |
|  | **NP08** | 60.5 | 1.6 | 23.63 | 66 | 1.21 | 1.13 | 1* | 0 | 0 | 0 |
|  | **NP09** | 58.1 | 1.61 | 22.41 | 67 | 1.22 | 1.33 | 1 | 0 | 1* | 0 |
|  | **NP10** | 75.9 | 1.6 | 29.65 | 55 | 1.47 | 1.32 | 1 | 0 | 1* | 0 |
|  | **NP11** | 80.4 | 1.63 | 30.26 | 57 | 1.04 | 1.13 | 1 | 0 | 1* | 0 |

## Material properties of the finite element (FE) model

The FE model of cartilage uses a fibril-reinforced poro-elastic (FRPE) material property^1,2^. This biphasic material simulates the interaction between the fluid phase of water, the solid phase of fibrils and porous non-fibrillar matrix representing proteoglycans. Therefore, the total stress tensor ( $\boldsymbol{\sigma}_{tot}$) consists of stresses of a compressive neo-Hookean non-fibrillar matrix ($\boldsymbol{\sigma}_{nf}$), fibrils ($\boldsymbol{\sigma}_{f}$) and fluid pressure (p):

| $\boldsymbol{\sigma}_{tot}=\boldsymbol{\sigma}_{nf}+ \sum_{i=1}^{4} {\boldsymbol{\sigma}_{f,p}}^{i}+\sum_{i=1}^{13} {\boldsymbol{\sigma}_{f,s}}^{i}-p\boldsymbol{I}$ | (S1) |
| --- | --- |

The stress of the compressive neo-Hookean non-fibrillar matrix is calculated as follows^3,4^:

| $\boldsymbol{\sigma}_{\mathrm{nf}}=\frac{1}{2}K_{\mathrm{nf}}\left( J\boldsymbol{-}\frac{1}{J} \right)\mathbf{I}+\frac{G_{\mathrm{nf}}}{J}\left( \mathbf{F}\mathbf{F}^{T}\boldsymbol{-}J^{\frac{\boldsymbol{2}}{\boldsymbol{3}}}\mathbf{I} \right)$ | (S2) |
| --- | --- |

where $\mathbf{I}$ is the identity tensor, $\mathbf{F}$ is the deformation gradient, J is the Jacobian of the deformation (determinant of the deformation gradient), $K_{\mathrm{nf}}$ and $G_{\mathrm{nf}}$ are bulk and shear moduli and are determined by the following equations:

| $K_{\mathrm{nf}}=\frac{E_{\mathrm{nf}}}{3\left( {1-2\nu}_{\mathrm{nf}} \right)}$ | (S3) |
| --- | --- |
| $G_{\mathrm{nf}}=\frac{E_{\mathrm{nf}}}{2\left( {1+\nu}_{\mathrm{nf}} \right)}$ | (S4) |

where $E_{nf}$ is the Young’s modulus and $\nu_{\mathrm{nf}}$ is the Poisson’s ratio of the non-fibrillar matrix (**Table S2**).

The fibrillar network at each integration point includes 4 primary and 13 secondary fibrils^2,5^. The stress of primary ($\boldsymbol{\sigma}_{\boldsymbol{f,p}}$) and secondary fibrils ($\boldsymbol{\sigma}_{\boldsymbol{f,s}}$) follow the following equations:

| $\boldsymbol{\sigma}_{\boldsymbol{f,p}}=C\boldsymbol{\sigma}_{\boldsymbol{f}}$ | (S5) |
| --- | --- |
| $\boldsymbol{\sigma}_{\boldsymbol{f,s}}=\boldsymbol{\sigma}_{\boldsymbol{f}}$ | (S6) |

where $\boldsymbol{\sigma}_{\boldsymbol{f}}$ is the calculated fibrillar strain and *C* is the ratio between primary and secondary fibrils.

The primary fibrils follow an arcade shape Benninghoff structure across the cartilage depth^6^, whose orientation is parallel to the articular surface in the superficial zone and gradually changes to perpendicular in the deep zone. On the contact surface, all primary fibrils orient towards the centre of the medial cartilage^5^. While the secondary fibrils randomly orient as described in xx. All fibrils have an non-linear elastic behaviour along the fibril direction^1^:

| $\sigma_{f}=\frac{1}{2}E_{f}^{\varepsilon}\varepsilon_{f}^{2}+E_{f}^{0}\varepsilon_{f}$ | (S7) |
| --- | --- |

Where $E_{f}^{0}$ and $E_{f}^{\varepsilon}$ are initial and strain-dependent fibril network moduli (**Table S2**), respectively, and $\varepsilon_{f}$ is the calculated fibril strain determined by:

| $\varepsilon_{f}=\left\{ \begin{matrix} \ln\left( \left\Vert\mathbf{F}\boldsymbol{e}_{f} \right\Vert\right) , when \varepsilon_{f}>0 \\ 0 , when \varepsilon_{f}>0 \end{matrix} \right.$ | (S8) |
| --- | --- |

Where $\boldsymbol{e}_{f}$ is the unit vector of fibril orientation.

The rate of fluid flow (*q*) is calculated based on Darcy’s law^7^:

| $q=-k\nabla p$ | (S9) |
| --- | --- |

Where ∇p is the fluid pressure gradient, and k is deformation-dependent permeability calculated by:

| $k=k_{0}\left( \frac{1+e}{1+e_{0}} \right)^{M}$ | (S10) |
| --- | --- |

where $k_{0}$ is the initial permeability (**Table S2**), *e* and $e_{0}$ are current and initial void ratios, respectively, and M is permeability void-ratio dependency constant (**Table S2**).

**Table S2.** Material parameters used in the FE^8^

| **Parameters** | | **Unit** | **Value** |
| --- | --- | --- | --- |
| $\boldsymbol{E}_{\boldsymbol{nf}}$ | Young’s moduli of non-fibrillar matrix | $MPa$ | 0.55 |
| $\boldsymbol{\nu}_{\mathbf{nf}}$ | Poisson' s ratio of non-fibrillar matrix | - | 0.36 |
| $\boldsymbol{E}_{\boldsymbol{f}}^{\boldsymbol{0}}$ | Initial fibril network moduli | $MPa$ | 0.65 |
| $\boldsymbol{E}_{\boldsymbol{f}}^{\boldsymbol{\varepsilon}}$ | Strain-dependent fibril network moduli | $MPa$ | 21.5 |
| $\boldsymbol{k}_{\boldsymbol{0}}$ | Permeability | ${10}^{-15}m^{4}N^{-1}s^{-1}$ | 0.89 |
| $\boldsymbol{M}$ | Permeability void-ratio dependency constant | - | 2.36 |

## Joint angles and moments


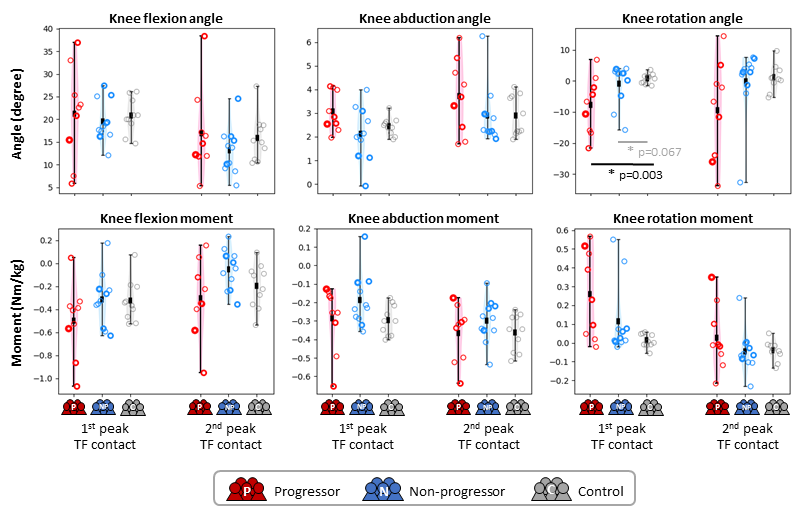


**Figure S1.** Top: Knee joint kinematic results; Bottom: Knee joint moments (Nm/kg) results at first and second peak knee joint loading for progressors (red), non-progressors (blue), and control (grey) groups. Individual subject means are shown by circles, with the thicker circles indicating a higher KL score at baseline. Dark squares indicate the group average, and vertical lines indicate the entire range. Stars * indicate a significant between-group differences (p<0.05 in black and 0.05<p<0.1 in grey) using the Mann Whitney U test with a p-value marked.

Differences in loading magnitude and location were accompanied with a more flexed, adducted and significantly more externally rotated knee in the OA progressors group compared to both non-progressors and controls group at the first peak and a more externally rotated knee at the second peak in progressors compared to both non-progressors and controls subjects (**Figure S1** top). However, knee moments including the abduction moment were not significantly different. (**Figure S1** bottom)

Hip angle and moments are illustrated in **Figure S2**. No significant differences were observed.


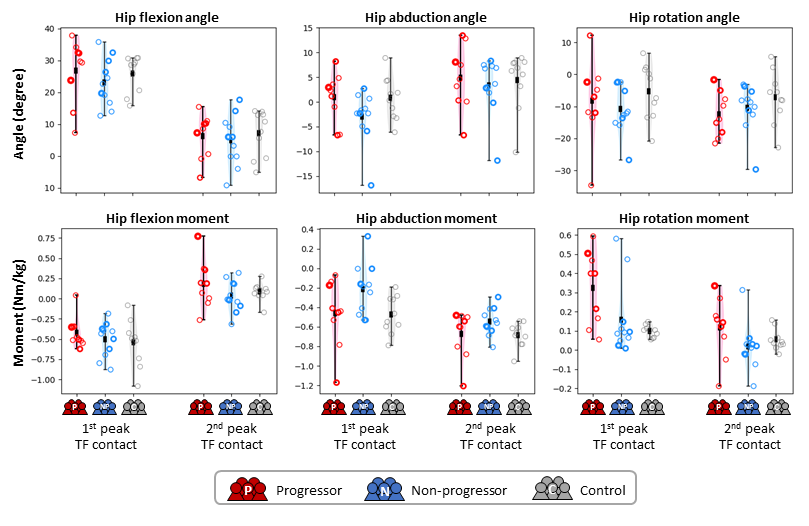


**Figure S2.** Top: Hip joint kinematic results; Bottom: Hip joint moments (Nm/kg) results at first and second peak knee joint loading for progressors (red), non-progressors (blue), and control (grey) groups. Individual subject averages are shown by circles where the weight of the circle indicates KL score at baseline with thicker circles indicating a higher KL score. No significant between group difference (p<0.05) was found.

## Joint contact of lateral compartment


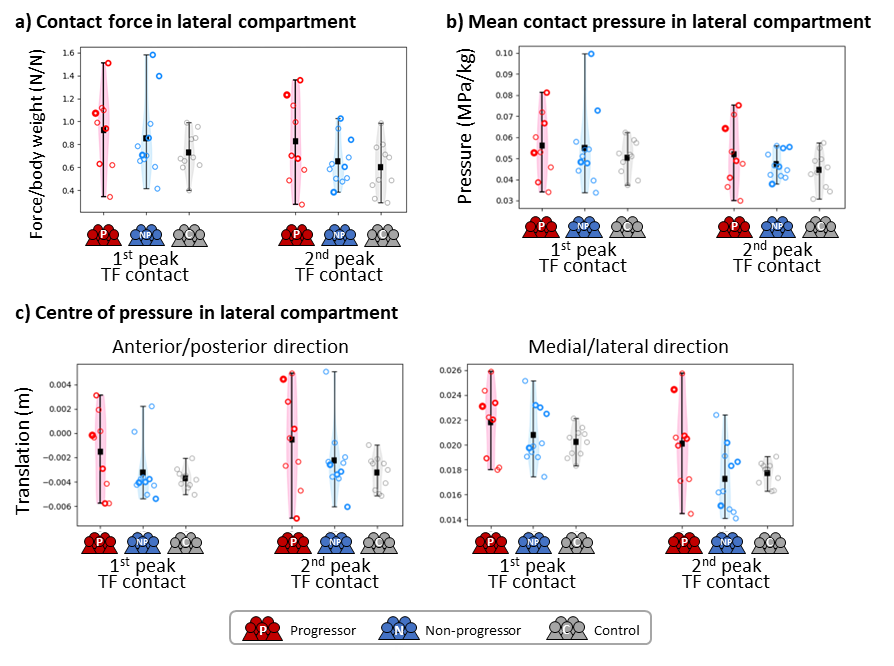


**Figure S3.** **a)** contact force in lateral compartment. **b)** Mean cartilage contact pressure of lateral compartment across all elements in contact; **c)** centre of pressure (COP) in the anterior/posterior (left), and medial/lateral (right) direction in the lateral compartment for OA progressors (red), non-progressors (blue) and control subjects (grey) at first and second peaks of TF contact forces. Individual subject means are shown by circles where the weight of the circle indicates KL score at baseline with thicker circles indicating a higher KL score. Dark squares indicate group average, and vertical lines indicate the full range.

Joint contact mechanics in the lateral compartment are illustrated in **Figure S3**. At both first and second peaks of tibio-femoral (TF) contact forces, progressors showed a more anterior-lateral centre of pressure (COP) compared with non-progressors and controls. However, no statistically significant differences were observed in lateral contact forces or mean contact pressures at either peak.

## Clustering results


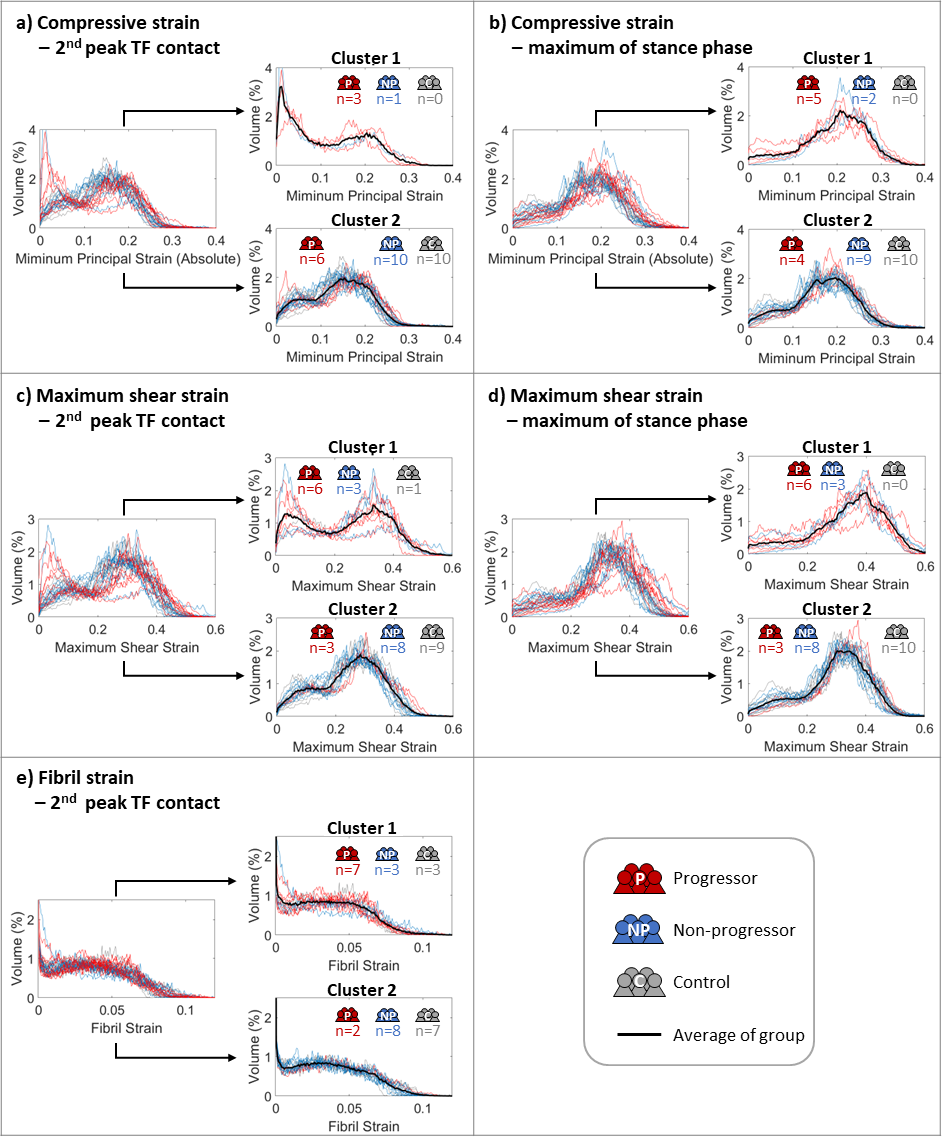


**Figure S4.** Unsupervised clustering results for histograms of key mechanical parameters, including **a)** absolute minimum principal strain at the second peak of TF contact forces, **b)** absolute minimum principal strain at the maximum value of the stance phase, **c)** maximum shear strain at the second peak of TF contact forces, **d)** maximum shear strain at the maximum value of the stance phase, and **e)** fibril strain the second peak of TF contact forces. Histograms of individual OA progressors, non-progressors and control subject are presented in red, blue and grey, respectively, with black lines indicating average values of the clustered groups.

**Table S3.** Clustering results for OA progressors (red), non-progressors (blue) and controls (grey) shown with KL scores. Clustering group 1 indicates the parameters are classified in the group dominated by progressors, whereas group 2 indicates parameters are classified in the group dominated by non-progressors and controls. Misclassified parameters are noted in red. Subjects for whom all, or all but one, parameters were misclassified are marked in red.

| **Subject** | | **KL**  **score** | **Clustering results** | | | | | | |
| --- | --- | --- | --- | --- | --- | --- | --- | --- | --- |
|  |  |  | **Minimum principal strain (absolute)** | | **Maximum shear strain** | | | **Fibril strain** | |
|  |  |  | **1^st^ peak** | **max** | **1^st^ peak** | **2^nd^ Peak** | **max** | **1^st^ peak** | **max** |
| **Progressors** | **P01** | KL1 | 2 | 2 | 2 | 1 | 2 | 2 | 2 |
|  | **P02** | KL2 | 1 | 1 | 1 | 1 | 1 | 1 | 1 |
|  | **P03** | KL2 | 1 | 2 | 1 | 2 | 1 | 1 | 2 |
|  | **P04** | KL1 | 1 | 1 | 1 | 1 | 1 | 1 | 1 |
|  | **P05** | KL3 | 1 | 1 | 1 | 1 | 1 | 1 | 2 |
|  | **P06** | KL1 | 1 | 1 | 1 | 1 | 1 | 1 | 1 |
|  | **P07** | KL1 | 2 | 2 | 2 | 2 | 2 | 2 | 2 |
|  | **P08** | KL1 | 2 | 2 | 2 | 1 | 2 | 1 | 1 |
|  | **P09** | KL1 | 1 | 1 | 1 | 2 | 1 | 1 | 1 |
| **Non-progressors** | **NP01** | KL2 | 2 | 2 | 2 | 1 | 1 | 2 | 2 |
|  | **NP02** | KL2 | 2 | 2 | 2 | 2 | 2 | 2 | 2 |
|  | **NP03** | KL1 | 2 | 2 | 2 | 2 | 2 | 2 | 2 |
|  | **NP04** | KL2 | 2 | 2 | 2 | 2 | 2 | 1 | 2 |
|  | **NP05** | KL1 | 2 | 2 | 2 | 2 | 2 | 2 | 2 |
|  | **NP06** | KL2 | 2 | 2 | 2 | 2 | 2 | 2 | 2 |
|  | **NP07** | KL1 | 1 | 1 | 1 | 1 | 1 | 1 | 1 |
|  | **NP08** | KL1 | 1 | 1 | 1 | 1 | 1 | 1 | 1 |
|  | **NP09** | KL1 | 2 | 2 | 2 | 2 | 2 | 2 | 2 |
|  | **NP10** | KL1 | 2 | 2 | 2 | 2 | 2 | 2 | 2 |
|  | **NP11** | KL1 | 2 | 2 | 2 | 2 | 2 | 2 | 2 |
| **Control** | **C01** | - | 2 | 2 | 2 | 2 | 2 | 2 | 2 |
|  | **C02** | - | 2 | 2 | 2 | 2 | 2 | 2 | 2 |
|  | **C03** | - | 2 | 2 | 2 | 2 | 2 | 2 | 2 |
|  | **C04** | - | 2 | 2 | 2 | 2 | 2 | 2 | 2 |
|  | **C05** | - | 2 | 2 | 2 | 2 | 2 | 2 | 2 |
|  | **C06** | - | 2 | 2 | 2 | 2 | 2 | 2 | 2 |
|  | **C07** | - | 2 | 2 | 2 | 2 | 2 | 2 | 2 |
|  | **C08** | - | 2 | 2 | 2 | 2 | 2 | 2 | 2 |
|  | **C09** | - | 2 | 2 | 2 | 2 | 2 | 2 | 2 |
|  | **C10** | - | 2 | 2 | 2 | 1 | 2 | 2 | 2 |

**Figure S4** shows the clustering results for key mechanical parameters, excluding the four best-performing ones illustrated in **Figure 6** in the main text. For minimum principal strain at the maximum of the stance phase (**Figure S4**b), and maximum shear strain at the first peak of TF contact (**Figure S4**c) and the maximum of the stance phase (**Figure S4**d), the identified cluster 1 was dominated by OA progressors (red), and cluster 2 by non-progressors (blue) and control (grey). The clustering accuracy of these parameters was comparable to the best four parameters mentioned in the main text (**Figure 6**), though 1 or 2 additional subjects were misclassified.

However, for the minimum principal strain at the second peak (**Figure S4**a), only 3 out of 9 progressors and 1 out of 11 non-progressors were grouped in cluster 1, with the remaining 26 subjects in cluster 2. For fibril strain at the second peak (**Figure S4**e), cluster 2 was dominated by non-progressors and controls, yet no domination of progressors was shown in cluster 1.

Clustering results for each individual subject are summarised in **Table S3**, which includes only the parameters where one cluster was dominated by progressors and the other by non-progressors and controls. Clustering was most accurate for controls, with all but one parameters of control subject correctly grouped into cluster 2 (dominated by non-progressors and controls), indicating that their mechanical responses served as a reliable reference for clustering. However, four subjects – P01, P07, NP07, and NP08 marked in red – were entirely misclassified into the opposite cluster, accounting for the majority of misclassifications.

### Reference

1. Ebrahimi, M. *et al.* Elastic, Viscoelastic and Fibril-Reinforced Poroelastic Material Properties of Healthy and Osteoarthritic Human Tibial Cartilage. *Ann. Biomed. Eng.* **47**, 953–966 (2019).

2. Wilson, W., Van Donkelaar, C. C., Van Rietbergen, B., Ito, K. & Huiskes, R. Stresses in the local collagen network of articular cartilage: A poroviscoelastic fibril-reinforced finite element study. *J. Biomech.* **37**, 357–366 (2004).

3. Hosseini, S. M., Wilson, W., Ito, K. & Van Donkelaar, C. C. A numerical model to study mechanically induced initiation and progression of damage in articular cartilage. *Osteoarthr. Cartil.* **22**, 95–103 (2014).

4. Wilson, W., Van Donkelaar, C. C., Van Rietbergen, B. & Huiskes, R. A fibril-reinforced poroviscoelastic swelling model for articular cartilage. *J. Biomech.* **38**, 1195–1204 (2005).

5. Mononen, M. E. *et al.* Effect of superficial collagen patterns and fibrillation of femoral articular cartilage on knee joint mechanics-A 3D finite element analysis. *J. Biomech.* **45**, 579–587 (2012).

6. Benninghoff, A. Form und Bau der Gelenkknorpel in ihren Beziehungen zur Funktion - Erste Mitteilung: Die modellierenden und formerhaltenden Faktoren des Knorpelreliefs. *Z. Anat. Entwicklungsgesch.* **76**, 43–63 (1925).

7. van der Voet, A. A comparison of finite element codes for the solution of biphasic poroelastic problems. *Proceedings of the Institution of Mechanical Engineers. Part H, Journal of engineering in medicine* vol. 211 209–211 at (1997).

8. Seyed Ali Elahi, Rocio Castro-Vinuelas, Anke Govaerts, R. L. Unconfined Compression Experimental Protocol for Cartilage Explants and Hydrogel Constructs: From Sample Preparation to Mechanical Characterization. in *Cartilage Tissue Engineering: An Introduction* 271–287 (2023). doi:10.1007/978-1-0716-2839-3_1.
